# 4,4′-(Hexane-1,6-diyldi­oxy)dianiline

**DOI:** 10.1107/S160053680901321X

**Published:** 2009-04-22

**Authors:** Muhammad Saif Ullah Khan, Zareen Akhter, Michael Bolte, Amna Siraj, Humaira M. Siddiqi

**Affiliations:** a Department of Chemistry, Quaid-i-Azam University, Islamabad 45320, Pakistan; b Institut für Anorganische Chemie, J. W. Goethe-Universität Frankfurt, Max-von-Laue-Strasse 7, 60438 Frankfurt/Main, Germany

## Abstract

The complete molecule of the title compound, C_18_H_24_N_2_O_2_, is generated by a crystallographic inversion centre. The torsion angles in the hexa­methyl­ene chain are consistent with an anti­periplanar conformation, whereas the conformation of the O—CH_2_—CH_2_—CH_2_ unit is *gauche*. The three-dimensional crystal packing is stabilized by N—H⋯O and N—H⋯N hydrogen bonding.

## Related literature

For aromatic diamines as building blocks for the preparation of high-performance polymers, see: Mehdipour-Ataei (2005 ▶; Mehdipour-Ataei *et al.* (2007 ▶). For the use of flexible linkages, see: Shao *et al.* (2007 ▶); Yin *et al.* (1998 ▶).
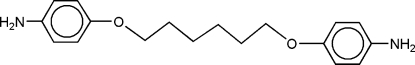


## Experimental

### 

#### Crystal data

C_18_H_24_N_2_O_2_
*M* *_r_* = 300.39
Orthorhombic, 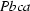

*a* = 5.4777 (6) Å
*b* = 13.6049 (12) Å
*c* = 21.7278 (18) Å
*V* = 1619.2 (3) Å^3^
*Z* = 4
Mo *K*α radiation
μ = 0.08 mm^−1^
*T* = 173 K
0.33 × 0.23 × 0.11 mm

#### Data collection

Stoe IPDS-II two-circle diffractometer
Absorption correction: none
9301 measured reflections
1854 independent reflections
1403 reflections with *I* > 2σ(*I*)
*R*_int_ = 0.056

#### Refinement

*R*[*F* ^2^ > 2σ(*F* ^2^)] = 0.039
*wR*(*F* ^2^) = 0.103
*S* = 1.02
1854 reflections
109 parameters
H atoms treated by a mixture of independent and constrained refinement
Δρ_max_ = 0.24 e Å^−3^
Δρ_min_ = −0.18 e Å^−3^

### 

Data collection: *X-AREA* (Stoe & Cie, 2001 ▶); cell refinement: *X-AREA*; data reduction: *X-AREA*; program(s) used to solve structure: *SHELXS97* (Sheldrick, 2008 ▶); program(s) used to refine structure: *SHELXL97* (Sheldrick, 2008 ▶); molecular graphics: *PLATON* (Spek, 2009 ▶) and *XP* (Sheldrick, 2008 ▶); software used to prepare material for publication: *SHELXL97*.

## Supplementary Material

Crystal structure: contains datablocks I, global. DOI: 10.1107/S160053680901321X/tk2416sup1.cif

Structure factors: contains datablocks I. DOI: 10.1107/S160053680901321X/tk2416Isup2.hkl

Additional supplementary materials:  crystallographic information; 3D view; checkCIF report

## Figures and Tables

**Table 1:** Hydrogen-bond geometry (Å, °)

*D*—H⋯*A*	*D*—H	H⋯*A*	*D*⋯*A*	*D*—H⋯*A*
N1—H1*A*⋯N1^i^	0.939 (18)	2.318 (18)	3.2248 (11)	162.1 (13)
N1—H1*B*⋯O1^ii^	0.899 (16)	2.318 (16)	3.1724 (14)	158.6 (13)

Symmetry codes: (i) 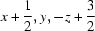; (ii) 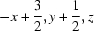.

**Fig. 1.**
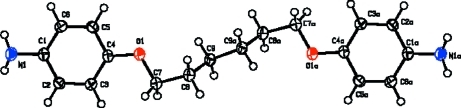
Perspective view of (I) showing the atom labelling scheme and displacement ellipsoids at the 50% probability level. H atoms are drawn as spheres of arbitrary radii. Unlabelled atoms are related by the symmetry operator 1 - x, -y, 1 - z.

## supplementary crystallographic information

### Comment

Aromatic diamines are valuable building blocks for the preparation of high-performance polymers including polyamides, polyimides and polyureas (Mehdipour-Ataei, 2005). Therefore, these can be used to produce desired alterations in the chemical nature of macromolecular chains (Mehdipour-Ataei *et al.*, 2007). Much research in recent years has focused on the design and synthesis of novel diamines in order to obtain suitable polymers. One of the popular approaches to achieve this goal is the introduction of flexible linkages such as an ether moiety (Shao *et al.*, 2007) and/or methylene spacers (Yin *et al.*, 1998) in the core structure of the diamines. These linkages increase the degree of freedom by reducing the segmental rotational barrier and inhibit close chain packing. The title compound, (I), in which flexible methylene spacers are present between the aryl ether moieties, is an outcome of efforts to modify the aromatic diamine monomers by flexible linkages in order to improve the processability and performance of the resulting polymers.

Molecules of (I) (Fig. 1) are located about a crystallographic centre of inversion. All torsion angles in the hexamethylene chain indicate an antiperiplanar conformation whereas the conformation of the O—CH_2_—CH_2_—CH_2_ unit is *gauche*. The crystal packing (Table 1) is stabilized by N—H···O and N—H···N hydrogen bonds which lead to a three-dimensional network.

### Experimental

The title compound (I) was synthesized in two steps. In the first step, bis(4-nitrophenoxy)hexane was prepared by Williamson's reaction. A three-neck round bottom flask equipped with Dean-Stark trap, thermometer, magnetic stirrer and nitrogen inlet was charged with a suspension of 1,6-hexane diol (2.25 g; 19.1 mmol) and anhydrous potassium carbonate (5.3 g; 38.2 mmol) in a mixture of N,*N*'-dimethyl formamide (DMF) (60 ml) and toluene (20 ml), and refluxed (at 403–408 K) for 2 h for azeotropic removal of water. After cooling to 343–343 K, 1-fluoro-4-nitro benzene (4.05 ml; 38.2 mmol) was added and the mixture was again refluxed for 6 h. Subsequently, some toluene was distilled off and the resulting mixture was poured into 500 ml of chilled water after cooling to room temperature. The crude product was filtered as yellow solid, washed thoroughly with water and recrystallized from ethanol to afford bis(4-nitrophenoxy)hexane. In the second step, a two-neck flask was charged with 1,6-bis(4-nitrophenoxy)hexane (2.5 g; 6.94 mmol), hydrazine monohydrate (10 ml), ethanol (80 ml) and 0.1 g of 5% palladium on carbon (Pd–C). The mixture was refluxed for 18 h and then filtered to remove the Pd–C. The filtrate was concentrated on rotary evaporator to remove the solvent and the resulting crude solid was recrystallized from ethanol to afford colourless crystals suitable for X-ray analysis, which were stored in air-tight glass bottles for further studies. Yield 72%; m.p. 414 K. Elemental analysis. Found C, 72.03, H, 7.90, N, 9.25. Calculated for C_18_H_24_N_2_O_2_: C, 71.97, H, 8.05, N, 9.33; IR (KBr pellet) in cm^-1^: 3395, 3311 (NH_2_), 1632 (N-H bending), 1385 (C-N stretching), 1233 (C-O-C), 2935 (C—H aliphatic), 3219 (C—H aromatic). ^1^H NMR (CDCl_3_) δ: 3.92 (s, 4H, NH_2_), 6.40 (d, 4H, J = 3.0 Hz), 6.70 (d, 4H, J = 2.9 Hz), 3.88 (t, 4H), 1.78 (m, 4H), 1.51 (m, 4H) p.p.m. ^13^C NMR (CDCl_3_) δ: 147.52 (2 C, C4), 139.83 (2 C, C1), 116.42 (4 C, C2,2'), 115.67 (4 C, C3,3'), 68.54 (2 C, C5), 29.38 (2 C, C6), 25.91 (2 C, C7) p.p.m.

### Refinement

H atoms bonded to C were geometrically positioned and refined using a riding model with with C—H(aromatic) = 0.95Å and *C*—*H*(methylene) = 0.99 Å, and with U(H) = 1.2 *U*_eq_(C). The H atoms bonded to N were freely refined, see Table 1 for distances.

### Crystal data

**Table:**

C_18_H_24_N_2_O_2_	*F*(000) = 648
*M**_r_* = 300.39	*D*_x_ = 1.232 Mg m^−^^3^
Orthorhombic, *P**b**c**a*	Mo *K*α radiation, λ = 0.71073 Å
Hall symbol: -P 2ac 2ab	Cell parameters from 6791 reflections
*a* = 5.4777 (6) Å	θ = 3.6–27.7°
*b* = 13.6049 (12) Å	µ = 0.08 mm^−^^1^
*c* = 21.7278 (18) Å	*T* = 173 K
*V* = 1619.2 (3) Å^3^	Plate, colourless
*Z* = 4	0.33 × 0.23 × 0.11 mm

### Data collection

**Table:**

Stoe IPDS-II two-circle diffractometer	1403 reflections with *I* > 2σ(*I*)
Radiation source: fine-focus sealed tube	*R*_int_ = 0.056
graphite	θ_max_ = 27.6°, θ_min_ = 3.5°
ω scans	*h* = −7→5
9301 measured reflections	*k* = −17→17
1854 independent reflections	*l* = −24→28

### Refinement

**Table:**

Refinement on *F*^2^	Secondary atom site location: difference Fourier map
Least-squares matrix: full	Hydrogen site location: inferred from neighbouring sites
*R*[*F*^2^ > 2σ(*F*^2^)] = 0.039	H atoms treated by a mixture of independent and constrained refinement
*wR*(*F*^2^) = 0.103	*w* = 1/[σ^2^(*F*_o_^2^) + (0.0626*P*)^2^] where *P* = (*F*_o_^2^ + 2*F*_c_^2^)/3
*S* = 1.02	(Δ/σ)_max_ = 0.001
1854 reflections	Δρ_max_ = 0.24 e Å^−^^3^
109 parameters	Δρ_min_ = −0.18 e Å^−^^3^
0 restraints	Extinction correction: *SHELXL97* (Sheldrick, 2008), Fc^*^=kFc[1+0.001xFc^2^λ^3^/sin(2θ)]^-1/4^
Primary atom site location: structure-invariant direct methods	Extinction coefficient: 0.016 (2)

### Special details

**Table:**

Geometry. All e.s.d.'s (except the e.s.d. in the dihedral angle between two l.s. planes) are estimated using the full covariance matrix. The cell e.s.d.'s are taken into account individually in the estimation of e.s.d.'s in distances, angles and torsion angles; correlations between e.s.d.'s in cell parameters are only used when they are defined by crystal symmetry. An approximate (isotropic) treatment of cell e.s.d.'s is used for estimating e.s.d.'s involving l.s. planes.
Refinement. Refinement of *F*^2^ against ALL reflections. The weighted *R*-factor *wR* and goodness of fit *S* are based on *F*^2^, conventional *R*-factors *R* are based on *F*, with *F* set to zero for negative *F*^2^. The threshold expression of *F*^2^ > σ(*F*^2^) is used only for calculating *R*-factors(gt) *etc*. and is not relevant to the choice of reflections for refinement. *R*-factors based on *F*^2^ are statistically about twice as large as those based on *F*, and *R*- factors based on ALL data will be even larger.

### Fractional atomic coordinates and isotropic or equivalent isotropic displacement parameters (Å^2^)

**Table:**

	*x*	*y*	*z*	*U*_iso_*/*U*_eq_	
N1	0.9705 (2)	0.55262 (7)	0.71082 (5)	0.0289 (3)	
H1A	1.131 (3)	0.5464 (11)	0.7257 (7)	0.045 (4)*	
H1B	0.956 (3)	0.6080 (11)	0.6885 (7)	0.044 (4)*	
O1	0.60683 (17)	0.20721 (5)	0.60327 (4)	0.0303 (2)	
C1	0.8839 (2)	0.46681 (8)	0.68123 (5)	0.0235 (3)	
C2	0.6800 (2)	0.47129 (8)	0.64298 (5)	0.0258 (3)	
H2	0.6060	0.5332	0.6351	0.031*	
C3	0.5818 (2)	0.38691 (8)	0.61598 (5)	0.0255 (3)	
H3	0.4432	0.3917	0.5899	0.031*	
C4	0.6879 (2)	0.29578 (8)	0.62748 (5)	0.0237 (3)	
C5	0.8897 (2)	0.29005 (8)	0.66663 (5)	0.0262 (3)	
H5	0.9604	0.2279	0.6755	0.031*	
C6	0.9879 (2)	0.37456 (8)	0.69278 (5)	0.0259 (3)	
H6	1.1270	0.3697	0.7187	0.031*	
C7	0.3984 (2)	0.20972 (8)	0.56312 (5)	0.0269 (3)	
H7A	0.4363	0.2478	0.5255	0.032*	
H7B	0.2582	0.2410	0.5841	0.032*	
C8	0.3376 (2)	0.10382 (8)	0.54656 (5)	0.0267 (3)	
H8A	0.1770	0.1024	0.5257	0.032*	
H8B	0.3227	0.0652	0.5850	0.032*	
C9	0.5262 (2)	0.05471 (7)	0.50505 (5)	0.0248 (3)	
H9A	0.6900	0.0621	0.5237	0.030*	
H9B	0.5282	0.0885	0.4647	0.030*	

### Atomic displacement parameters (Å^2^)

**Table:**

	*U*^11^	*U*^22^	*U*^33^	*U*^12^	*U*^13^	*U*^23^
N1	0.0355 (7)	0.0239 (5)	0.0274 (5)	−0.0043 (4)	−0.0043 (5)	−0.0005 (4)
O1	0.0369 (6)	0.0204 (4)	0.0336 (5)	−0.0005 (3)	−0.0102 (4)	−0.0031 (3)
C1	0.0284 (6)	0.0230 (5)	0.0192 (5)	−0.0030 (4)	0.0032 (5)	−0.0002 (4)
C2	0.0311 (7)	0.0213 (5)	0.0251 (6)	0.0025 (5)	−0.0006 (5)	0.0002 (4)
C3	0.0271 (6)	0.0256 (6)	0.0238 (5)	0.0003 (4)	−0.0023 (5)	0.0001 (4)
C4	0.0277 (7)	0.0209 (5)	0.0226 (5)	−0.0018 (4)	0.0012 (5)	−0.0021 (4)
C5	0.0295 (7)	0.0222 (5)	0.0269 (6)	0.0036 (4)	0.0000 (5)	0.0001 (4)
C6	0.0249 (6)	0.0287 (6)	0.0241 (5)	0.0004 (5)	−0.0025 (5)	0.0000 (4)
C7	0.0283 (7)	0.0248 (6)	0.0275 (6)	0.0012 (5)	−0.0018 (5)	−0.0031 (4)
C8	0.0277 (7)	0.0253 (6)	0.0271 (6)	−0.0030 (4)	0.0004 (5)	−0.0025 (4)
C9	0.0259 (6)	0.0234 (6)	0.0250 (5)	−0.0041 (4)	−0.0005 (5)	−0.0015 (4)

### Geometric parameters (Å, °)

**Table:**

N1—C1	1.4147 (14)	C5—C6	1.3908 (16)
N1—H1A	0.939 (18)	C5—H5	0.9500
N1—H1B	0.899 (16)	C6—H6	0.9500
O1—C4	1.3879 (13)	C7—C8	1.5218 (15)
O1—C7	1.4372 (15)	C7—H7A	0.9900
C1—C2	1.3937 (17)	C7—H7B	0.9900
C1—C6	1.4011 (15)	C8—C9	1.5258 (16)
C2—C3	1.3968 (15)	C8—H8A	0.9900
C2—H2	0.9500	C8—H8B	0.9900
C3—C4	1.3919 (16)	C9—C9^i^	1.532 (2)
C3—H3	0.9500	C9—H9A	0.9900
C4—C5	1.3966 (17)	C9—H9B	0.9900
			
C1—N1—H1A	113.3 (10)	C5—C6—H6	119.7
C1—N1—H1B	114.7 (10)	C1—C6—H6	119.7
H1A—N1—H1B	110.0 (14)	O1—C7—C8	107.15 (9)
C4—O1—C7	117.63 (8)	O1—C7—H7A	110.3
C2—C1—C6	118.16 (10)	C8—C7—H7A	110.3
C2—C1—N1	120.25 (10)	O1—C7—H7B	110.3
C6—C1—N1	121.43 (11)	C8—C7—H7B	110.3
C1—C2—C3	121.54 (10)	H7A—C7—H7B	108.5
C1—C2—H2	119.2	C7—C8—C9	113.96 (10)
C3—C2—H2	119.2	C7—C8—H8A	108.8
C4—C3—C2	119.72 (11)	C9—C8—H8A	108.8
C4—C3—H3	120.1	C7—C8—H8B	108.8
C2—C3—H3	120.1	C9—C8—H8B	108.8
O1—C4—C3	124.86 (11)	H8A—C8—H8B	107.7
O1—C4—C5	115.83 (9)	C8—C9—C9^i^	112.53 (12)
C3—C4—C5	119.31 (10)	C8—C9—H9A	109.1
C6—C5—C4	120.58 (10)	C9^i^—C9—H9A	109.1
C6—C5—H5	119.7	C8—C9—H9B	109.1
C4—C5—H5	119.7	C9^i^—C9—H9B	109.1
C5—C6—C1	120.67 (11)	H9A—C9—H9B	107.8
			
C6—C1—C2—C3	−0.75 (17)	C3—C4—C5—C6	−1.58 (18)
N1—C1—C2—C3	−176.24 (11)	C4—C5—C6—C1	1.14 (18)
C1—C2—C3—C4	0.31 (18)	C2—C1—C6—C5	0.03 (17)
C7—O1—C4—C3	0.28 (17)	N1—C1—C6—C5	175.46 (10)
C7—O1—C4—C5	179.52 (10)	C4—O1—C7—C8	−176.37 (9)
C2—C3—C4—O1	−179.93 (11)	O1—C7—C8—C9	−69.25 (12)
C2—C3—C4—C5	0.85 (17)	C7—C8—C9—C9^i^	173.85 (12)
O1—C4—C5—C6	179.13 (10)		

Symmetry codes: (i) −*x*+1, −*y*, −*z*+1.

### Hydrogen-bond geometry (Å, °)

**Table:**

*D*—H···*A*	*D*—H	H···*A*	*D*···*A*	*D*—H···*A*
N1—H1A···N1^ii^	0.939 (18)	2.318 (18)	3.2248 (11)	162.1 (13)
N1—H1B···O1^iii^	0.899 (16)	2.318 (16)	3.1724 (14)	158.6 (13)

Symmetry codes: (ii) *x*+1/2, *y*, −*z*+3/2; (iii) −*x*+3/2, *y*+1/2, *z*.
